# 

**Published:** 1866-06

**Authors:** 


### CONTENTS.

ORIGINAL COMMUNICATIONS.

Filling Teeth,........................................ 239
Dental Education,........................................ 242
Dental Education as Connected with Dental Associations,........................................ 246
Nitrous Oxide for Dental Anæsthesia,........................................ 249
External Periostitis,........................................ 251

PROCEEDINGS OF SOCIETIES.

Proceedings of the Central States Dental Association,........................................ 252
Discussions of Central States Dental Association,........................................ 259

SELECTIONS.

On the Action of Medicinal Preparations of Iron on the Teeth,........................................ 270
Operation for Hare-Lip, &c........................................ 272
Sulphate of Bebeeria in Facial Neuralgia,........................................ 273
Syphilitic Sarcocele. Sloughing of the Scrotum. Removal of the Gland. Nitrous-Oxide. 273
The Agassiz Expedition,........................................ 274

CORRESPONDENCE,........................................ 274

EDITORIAL.

The Call,........................................ 280
To the Dentists of Ohio,........................................ 280
Measuring Cup,........................................ 281
American Dental Association,........................................ 282
The American Dental Convention........................................ 282
Central States Dental Association,........................................ 283
Automatic Plugger,........................................ 283
Bradley's Lip and Cheek Holder,........................................ 284
A File Carrier,........................................ 284

